# Microbiome-Metabolomics Analysis Reveals the Protection Mechanism of *α*-Ketoacid on Adenine-Induced Chronic Kidney Disease in Rats

**DOI:** 10.3389/fphar.2021.657827

**Published:** 2021-05-11

**Authors:** Yenan Mo, Huang Sun, Lei Zhang, Wenjia Geng, Lixin Wang, Chuan Zou, Yuchi Wu, Chunlan Ji, Xusheng Liu, Zhaoyu Lu

**Affiliations:** ^1^ The Second Clinical College, Guangzhou University of Chinese Medicine, Guangzhou, China; ^2^ Department of Emergency, TCM-Integrated Hospital, Southern Medical University, Guangzhou, China; ^3^ State Key Laboratory of Dampness Syndrome of Chinese Medicine, Nephrology Department, The Second Affiliated Hospital of Guangzhou University of Chinese Medicine, Guangzhou, China

**Keywords:** chronic kidney disease, *α*-ketoacid, metabolic profiles, microbiota, reno-protective

## Abstract

**Objectives:** As nitrogen-free precursors of corresponding essential amino, *α*-ketoacid have been widely prescribed to end-stage renal disease patients together with a low protein diet However, the impact of *α*-ketoacid on intestinal microbiota in chronic kidney disease (CKD) individuals is unknown. The study aims at investigating the variation in the intestinal microbiota and metabolic profile in response to *α*-ketoacid treatment in an adenine-induced CKD rat model.

**Design:** Rats in the treatment groups were given solution of compound *α*-ketoacid tablets. At the end of the study, blood, feces, colon tissues and kidney tissues were collected and processed for biochemical analyses, histological and western blot analyses, 16S rRNA sequence and untargeted metabolomic analyses.

**Results:** *α*-Ketoacid treatment reduced serum creatinine, blood urea nitrogen and 24 h urine protein, and alleviated tubular atrophy, glomerulosclerosis and interstitial fibrosis in adenine-induced CKD rats. Moreover, *α*-ketoacid significantly improved intestinal barrier and increased the abundance of *Methanobrevibacter, Akkermansia*, *Blautia and Anaerositipes* while reduced the abundance of *Anaerovorax* and *Coprococcus_3* at the genus level. In addition, our results also demonstrated that *α*-ketoacid significantly reduced the concentrations of indoxyl sulfate, betaine, choline and cholesterol. Spearman’s correlation analysis revealed that the abundance of Coprococcus_3 was positively correlated with serum level of betaine, trimethylamine N-oxide, indoxyl sulfate, cholic acid and deoxycholic acid.

**Conclusion:** *α*-Ketoacid has a reno-protective effect against adenine-induced CKD, which may be mediated regulation of serum metabolic profiles via affecting intestinal microbial community.

## Introduction

Chronic kidney disease (CKD) is an emerging epidemic that contributes to high incidences of morbidity and mortality (Collaboration, 2020). CKD results in changes in the structural and functional changes of kidney in CKD patients, eventually leading to end-stage kidney disease (ESKD) that requires renal replacement treatments. Therefore, an effective intervention to prevent progressive CKD is urgent.

α-Ketoacid have been widely prescribed to ESKD patients together with a low protein diet (LPD) (Mitch, 2000; Bernhard et al., 2001), which has been indicated by guidelines to be a fundamental tool to delay CKD progressions (Group, 2013). Most *α*-ketoacid supplementations contain four ketoacid (isoleucine, leucine, phenylalanine and valine), one hydroxyacid (methionine), and four essential amino acids (tryptophan, threonine, histidine and tyrosine). As nitrogen-free precursors of corresponding essential amino, *α*-ketoacid can transfer an amino group to a ketoacid to form a new amino acid, then *α*-ketoacid can improve protein status without providing nitrogen products while re-using available nitrogen in CKD individuals. Besides increasing body weight and muscle body mass, *α*-ketoacid showed many protective effect on CKD individuals, including reducing proteinuria and histological damage of kidney (Gao et al., 2010), reducing phosphate and parathyroid hormone levels (Garneata et al., 2016; Di Iorio et al., 2018), and declining diastolic blood pressure (Di Iorio et al., 2018) when supplemented with protein restriction in CKD patients and nephrectomy animals. However, the impact of intestinal function and intestinal microbiota with the presence of *α*-ketoacid is unknown.

Emerging studies have reported the relationship between CKD development and microbial composition and function changes in the intestines (Meijers and Evenepoel, 2011; Nallu et al., 2017; Wang et al., 2019). These effects have partly been proven to be mediated through metabolites. Gut microbiota may be the origin of the abnormal serum metabolites previously associated with CKD and its complications. Therefore, in order to characterize the global effects of *α*-ketoacid on CKD, it is interesting to combine research into gut microbiota with metabolomics.

In this study, we aimed to explore the beneficial effects of *α*-ketoacid on CKD, as well as its potential mechanisms of action, in adenine-induced CKD rats. The characteristics of CKD and effects of *α*-ketoacid treatment were evaluated according to blood biochemical indexes, and renal and colon pathological changes. Integrating serum untargeted metabolomics and sequencing of gut microbiota was used to investigate the response to *α*-ketoacid treatment in adenine-induced CKD rats. we revealed that several feature gut microbiota and metabolic pathways were highly associated with the beneficial outcomes of CKD. Our study showed the efficacy of *α*-ketoacid that could be considered as a promising strategy to treat CKD in clinical practice, not only as a prescribed medicine for nutrition management.

## Materials and Methods

### Experimental Animals and Medicinal Intervention

Sprague–Dawley rats (Male, weight: 180–220 g, grade: specific pathogen-free) were purchased from the Laboratory Animal Center of Southern Medical University (Guangzhou, China). Under a 12-h light/dark cycle, the rats were housed in a specific pathogen-free animal breeding room in Guangdong Provincial Hospital and given free access to water. All experiments were evaluated and approved by the Ethics Committee of Animal Experiments, Guangdong Provincial Hospital of Chinese Medicine (approval no. 2020039).

Rats were randomly assigned into the Sham Group (*n* = 8) and the CKD Group (*n* = 16) with random number generated by SPSS. Sham rats were fed standard rat chow (containing 18% protein, 58% Carbohydrate and 4.5% fat) and CKD rats were fed standard rat chow containing 0.75% w/w adenine for 4 weeks. Then serum creatinine (SCR) was measured to confirm whether the adenine-induced CKD model was set successfully. CKD rats were divided randomly into the Adenine Group (*n* = 8) and *α*-ketoacid Group (*n* = 8) with consideration of serum creatinine (SCR). *α*-Ketoacid-treated rats were given solution of compound *α*-ketoacid tablets (1.6 g/kg/day, Fresenius-Kabi, Lot No: 81NK302, Bad Homburg, Germany) by intragastric administration once per day for eight weeks. Sham Group and Adenine Group were administrated with the same volume of normal saline. Body weight were recorded once a week throughout the experiment. After four-week intervention, metabolic cages were used to collect 24 h urine for urinary protein excretion (24 h Upro) testing. Blood pressure was performed by rat tail blood pressure analysis system (BP-2010A; Softron, Tokyo, Japan). At the end point of the experiment, rats were given an intraperitoneal injection of 2.0% pentobarbital sodium (30 mg/kg body weight) for anesthetization. When rats became unconscious, they were euthanized using cervical dislocation. Blood, feces, kidney and colon tissues were collected for 16S rRNA sequence, untargeted metabolomic, histological and western blot analyses.

### Biochemical Analysis

SCR and blood urea nitrogen (BUN) were measured by Cobas C702 automatic analyzers (Roche, Basel, Switzerland). Proteinuria was measured using a bicinchoninic acid protein detection kit (Thermo Fisher Scientific, Waltham, MA, United States). Serum level of diamine oxidase (DAO) were measured in accordance with the enzyme-linked immunosorbent assay kit instructions (Cusabio, Wuhan, China).

### Histological Analysis

Cross-sections of the kidney and colon tissues of rats were fixed with 10% buffered paraformaldehyde, and then dehydrated and embedded in paraffin. Paraffin-embedded tissues were cut into 3-µm sections and used for histological and immunohistochemical analysis. The colon Sections were stained with hematoxylin and eosin (HE) staining (Boster Bio, Wuhan, China). The kidney sections were stained with periodic acid–Schiff (PAS) and Masson’s trichrome stains (Nanjing Jiancheng, Jiangsu, China). Abnormal parenchyma was recognized be the presence of one or more of the following: tubular inflammation (tubulitis), tubular dilatation or dropout, interstitial inflammation and or fibrosis. Quantitative scoring was performed as follows: The extent of abnormal (inflamed) renal parenchyma was visually estimated as a percentage of the total cortical area, in well-oriented sections which included both the renal cortex and medulla (Lobel et al., 2020).

### Immunohistochemistry

Paraffin-embedded rat kidney slides were deparaffined, rehydrated, and immersed in 3% hydrogen peroxide for 10 min at room temperature to block endogenous peroxidase activity. All sections were heated in Tris-EDTA buffer (pH 9.0, Boster, Wuhan, China), blocked with 5% blocking buffer for 30min at 37°C, incubated with primary antibodies against fibronectin (FN) (1:100, Abcam Cat# ab2413, Cambridge, England), type I collagen (Col-I) (1:100, Abcam Cat# ab254113, Cambridge, England) at 4°C overnight, incubated with species-specific secondary antibody (SV0004, Boster, Wuhan, China), developed with 3,3′-diaminobenzine (DAB, Invitrogen, California, United States) and counterstained with hematoxylin. The integrated optical density (iod) values of the positive staining areas were measured by ImagePro Plus 6.0 software (Media Cybernetics, CA, United States).

### Western Blotting Analysis

Kidney cortex and colon tissues were lyzed in 1 ml radioimmunoprecipitation assay lysis buffer containing 1 mM phenylmethyl sulfonyl fluoride and 1% phosphatase inhibitor cocktail (Thermo Fisher Scientific). A bicinchoninic acid protein detection kit was used to detect protein concentrations. Protein samples (50 μg) were boiled with sodium dodecyl sulfate polyacrylamide gel electrophoresis (SDS-PAGE) loading buffer, then electrophoresed on a 10% polyacrylamide gel under denaturing conditions and wet-transferred to polyvinylidene difluoride membranes (Millipore, Burlington, MA, United States). Membranes were exposed to blocking buffer for 2 h and hybridized with primary antibody against ZO-1 (1:1,000; Abcam Cat# ab96587, Cambridge, England), Occludin (1:1,000; Abcam Cat# ab168986, Cambridge, England), Claudin-1 (1:500; Abcam Cat# ab15098, Cambridge, England), IL-22 (1:1,000, Abcam Cat# ab5984, Cambridge, England) or *ß*-actin (1:2,000; Cell Signaling Technology Cat# 4970, Boston, United States) overnight at 4°C, followed by horseradish peroxidase-labeled anti-rabbit IgG (1:3,000; Cell Signaling Technology Cat# 7074, Boston, United States) at room temperature. Membranes were washed and then visualized using an enhanced chemiluminescence detection system (Bio-Rad, Hercules, CA, United States). Image Lab System (Bio-Rad) was used to captured and analyzed the signals.

### Processing of 16S rRNA Gene Sequences

#### Sample Collection

At Week 8, fresh feces of rats from each group were collected in centrifuge tubes, placed into empty sterilized microtubes, put into liquid nitrogen and stored at −80°C within 1 h after collection.

#### DNA Extraction and Amplicon Generation

E.Z.N.A. ® Soil DNA Lit (Omega Bio-tek, Norcross, GA, United States) was used for genomic DNA extraction according to manufacturer’s protocols. The concentrations and purity of extracted DNA were measured by a NanoDrop (NanoDrop ND-2000, United States). The 16S rRNA gene was amplified by PCR with a forward primer 341F (5-CCTACGGGNGGCWGCAG-3) and a reverse primer 806R (5-GGACTACHVGGGTATCTAAT-3) targeting the V3–V4 region of the 16S rRNA gene of bacteria. PCR products were purified using the AxyPrep DNA Gel Extraction Kit (Axygen Biosciences, Union City, CA, United States) according to the manufacturer’s instructions and quantified using QuantiFluor™-ST (Promega, United States). The PCR products of different samples were mixed equally and subsequently used to construct Illumina library. Then, the amplicon library was paired end sequenced (2 × 250) on an Illumina PE2500 platform (Illumina, SanDiego, United States) according to the standard protocols. After DNA from feces was extracted, 16S rRNA 132 genes of distinct regions (V3-V4) were amplified with specific primer (341F-806R).

#### Library Preparation and Sequencing

Quality filtering was done with Trimmomatic (version 0.33) to extract clean reads. Overlapping paired-end reads were merged to tags by FLASH (version 1.2.11), then clustered to operational taxonomic units (OTUs) at 97% sequence similarity by USEARCH UPARSE (version 9. in2.64). Taxonomic ranks were assigned to OTU representative sequences on the database Greengene (version gg_13_5). Alpha diversity (Ace and Chao index) was performed to reflect the community richness with QIIME (version 1.9.1). For beta diversity, principal coordinate analysis (PCoA) based on unweighted unifrac was calculated at the OTU level for 16S rDNA gene sequencing using the vegan package (version 2.5.3). The relative abundances of differential bacteria in the gut microbiota at phylum level and genus level was analyzed and presented by stacked bar. After unclassified microbiota were removed, F/B ratio was calculated as the ratio between the intestinal abundance of *Firmicutes* and that of *Bacteroidetes* at the genus level. Welch’s T text was used to identify features that differed significantly between groups. Functional classification schemes of KEGG (Kyoto Encyclopedia of Genes and Genomes) Orthology were predicted by Tax4Fun (version 1.0). All statistical analyses were performed using R software (version 3.5.0; R Foundation for Statistical Computing, Vienna, Austria).

### UPLC–MS Analysis for Metabolomics

Liquid chromatography-tandem mass spectrometry (LC-MS/MS) analyses were performed using an Ultra-high performance liquid chromatography (UHPLC) system (1,290, Agilent Technologies) with a UPLC HSS T3 column (2.1 mm × 100 mm, 1.7 μm) coupled to Q Exactive (Orbitrap MS, Thermo). The metabolomic procedure, including instruments and regents, metabolite extraction, data preprocessing and annotation, statistical analysis, were descripted detailly in Supplementary Item S1.

### Statistics Analysis

SPSS software (version 18.0, SPSS, Inc., Chicago, IL, United States) was used for statistical analysis. All data with normal distributions are presented as the mean ± standard error of the mean. For kidney and colon outcomes, differences among three groups were determined by one-way analysis of variance, followed by the Tukey test. For results on microbiota and metabolites, the difference between Sham Group and Adenine Group as well as between Adenine Group and *α*-Ketoacid Group were determined by Welch’s T text. Differences were considered statistically significant at *p* < 0.05.

## Results

### *α*-Ketoacid Alleviated Renal Failure Phenotypes in Adenine-Induced Chronic Kidney Disease Rats

**FIGURE 1:**
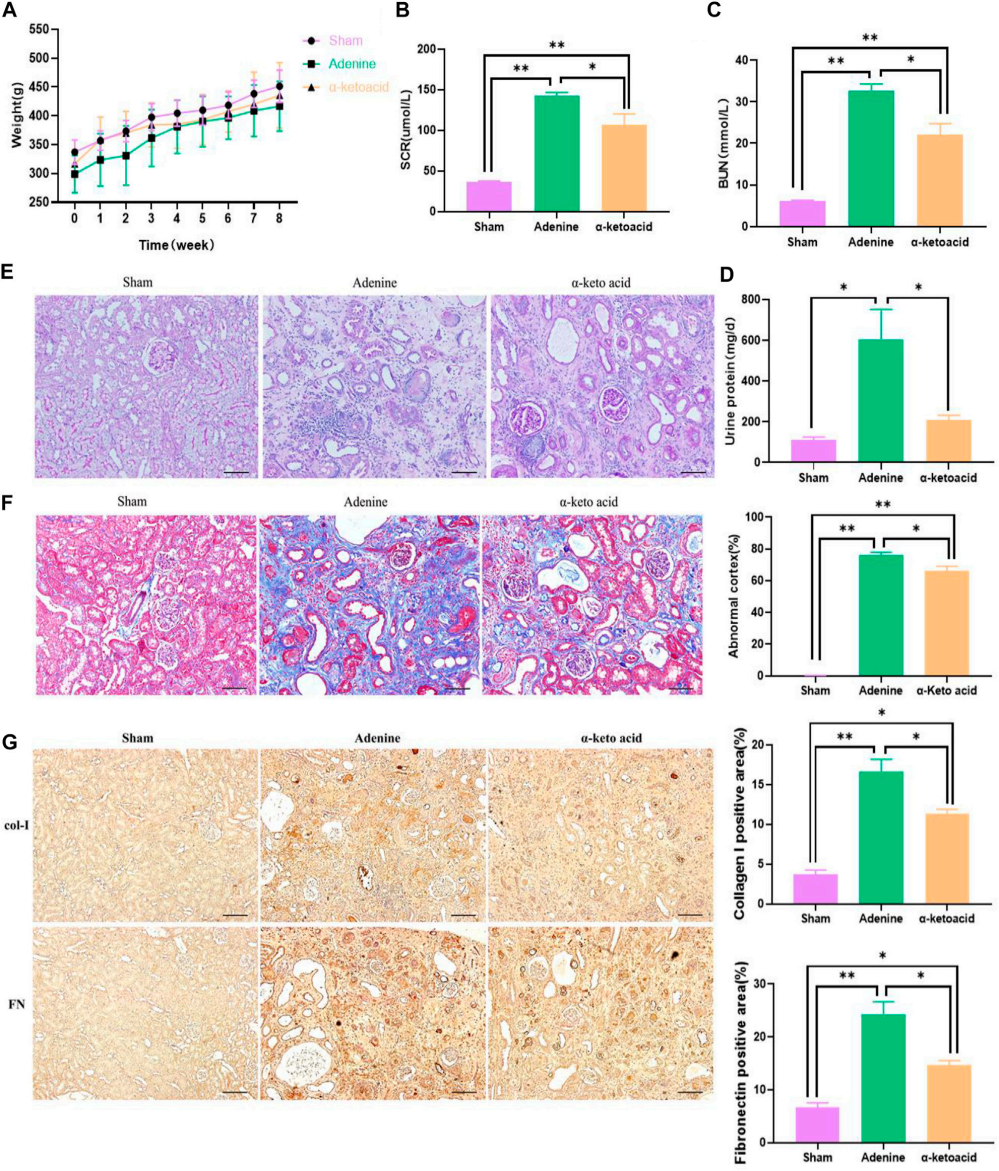
*α*-Ketoacid alleviated renal failure phenotypes in adenine-induced CKD rats. **(A)** Body weight. **(B)** Serum creatinine. **(C)** Blood urea nitrogen. **(D)** Twenty-four-hour urinary protein quantitation. **(E)** PAS staining of kidney tissue. **(F)** Masson staining of kidney tissue. **(G)** Immunohistochemical staining of Collagen I and Fibronectin expression. Data are presented as the means ± standard error of the mean, *n* = 8 rats per group (*p < 0.05, **p < 0.001).

We first evaluated the protective effect of *α*-ketoacid on CKD. It is common for CKD individuals to suffer from losing weight, *α*-ketoacid treatment seems to help CKD rats to gain weight compared with Adenine Group, even though there is no significant difference among groups (Figure 1A). BUN, SCR level and 24 h urine protein in Adenine Group were significantly increasing than that in Sham Group (*p* < 0.0001 for SCR, *p* < 0.0001 for BUN, *p* = 0.002 for 24 h-Upro) and decreased in *α*-ketoacid-treated group (*p* = 0.015 for SCR; *p* = 0.001 for BUN; *p* = 0.016 for 24 h-Upro) (Figures 1B–D). No differences were observed between groups in blood albumin, as well as blood pressure, including systolic blood pressure (SBP) and diastolic blood pressure (DBP) (Supplementary Figure S1).

In accord with the improved renal function, changes of histopathological were improved by KA. Renal tissue PAS and Masson staining showed enlarged renal tubular lumen, mononuclear lymphocyte infiltration, renal tubular atrophy, and interstitial fibrosis in Adenine Group (Figures 1E,F). Through Masson staining, histology-based renal injury scores in *α*-ketoacid Group were lower than that in Adenine Group. Tubulointerstitial fibrosis (TIF) is the common final pathway for CKD progression to ESRD. Immunohistochemistry staining showed that TIF markers Collagen Ⅰ and Fibronectin were significantly increased in the kidney of adenine-induced CKD rats and were strikingly inhibited after *α*-ketoacid administration (Figure 1G).

### α-Ketoacid Improved the Intestinal Barrier Integrity in Adenine-Induced Chronic Kidney Disease Rats

**FIGURE 2:**
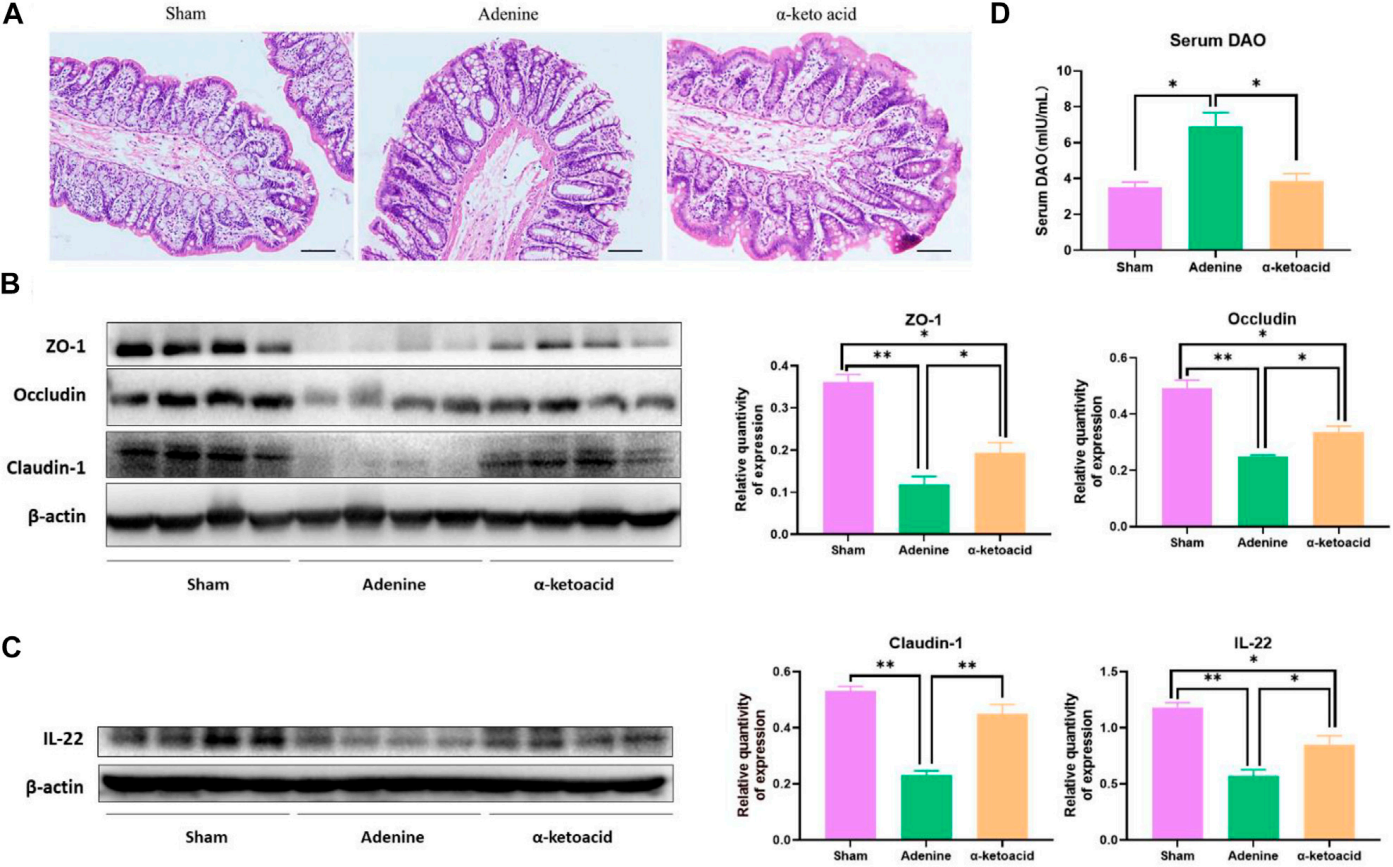
*α*-Ketoacid improved the intestinal barrier integrity in adenine-induced CKD rats. **(A)** Hematoxylin and eosin staining of colon tissue. **(B)** The expression of tight junction protein of the intestinal mucosal barrier (occludin, claudin-1, and ZO-1) in colon tissue. **(C)** The expression of IL-22 in colon tissue. **(D)** Serum level of diamine oxidase. Data are presented as the means ± standard error of the mean, *n* = 8 rats per group (*p < 0.05, **p < 0.001).

Compared with Sham Group, HE staining of colon tissue showed obvious edema in the mucosal layer and lamina propria in Adenine Group. In addition, obvious infiltration of lymphocyte monocytes was observed in the mucosal layer of adenine-induced CKD rats. After *α*-ketoacid intervention, the edema in the mucosal layer and lamina propria was significantly improved, and the infiltration of inflammatory cells in the mucosal layer was reduced (Figure 2A). The expression of IL-22 and tight junction protein of the intestinal mucosal barrier (ZO-1, occluding and claudin-1) in colon tissues was significantly decreased in Adenine Group, while this expression was obviously higher in *α*-ketoacid Group (Figures 2B,C). Compared with Sham Group, Adenine Group had a significantly higher prevalence of elevated DAO in serum, while the level of serum of DAO was significantly lower after *α*-ketoacid treatment (Figure 2D). These results collectively suggested intestinal barrier injury in CKD rats and *α*-ketoacid could repair the injury of intestinal barrier integrity.

### Regulation of *α*-Ketoacid on gut Microbiome in Adenine-Induced Chronic Kidney Disease Rats

**FIGURE 3:**
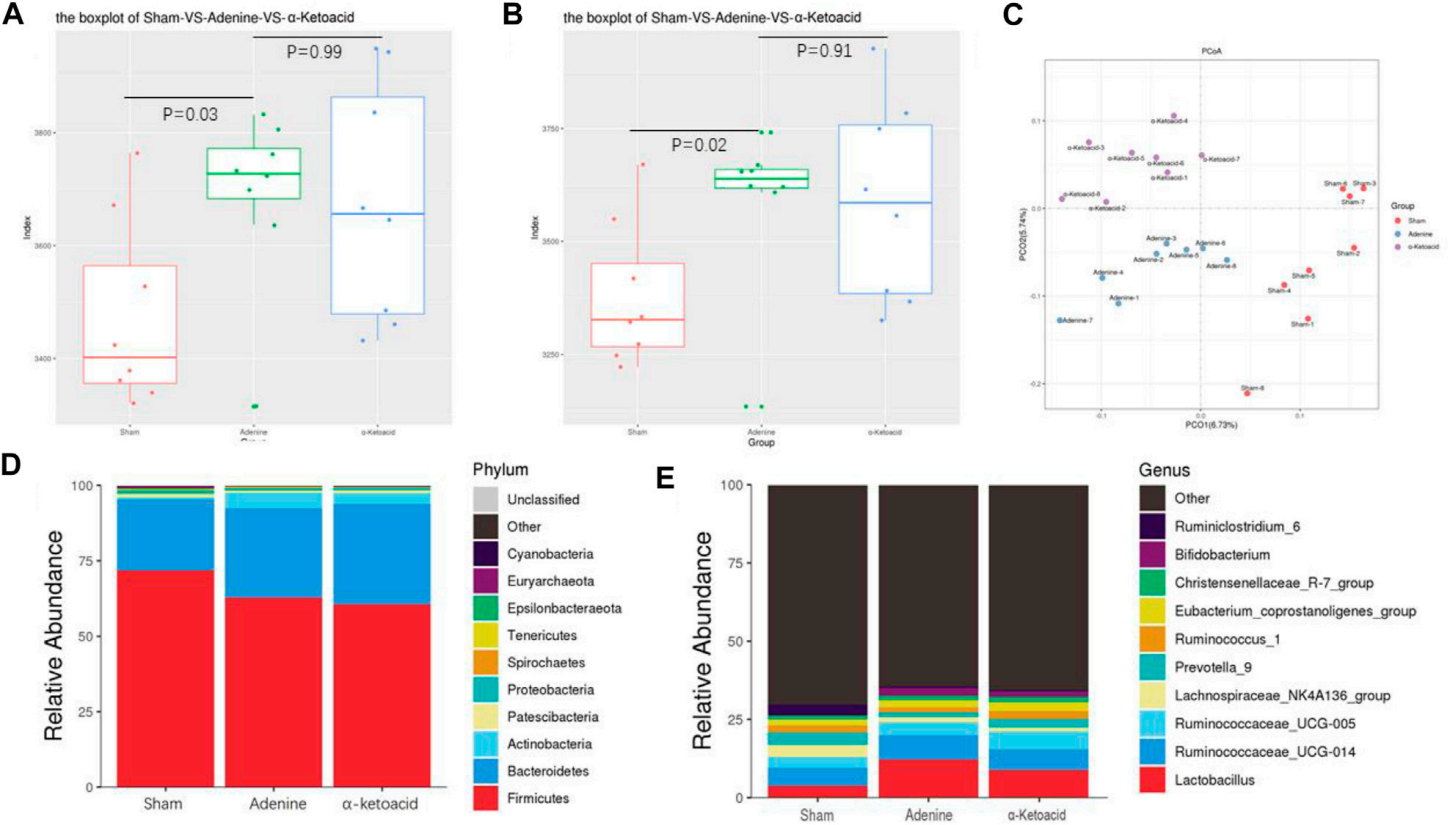
Regulation of α-ketoacid on gut microbiome in adenine-induced CKD rats. **(A)** The chao index of *α*-diversity. **(B)** The ace index of *α*-diversity. **(C)** PCoA plots based on unweighted unifrac to compare *β*-diversity of gut microbiota. **(D)** Compositions of gut microbiota at the phylumlevel. **(E)** Compositions of gut microbiota at the genus level.

**FIGURE 4:**
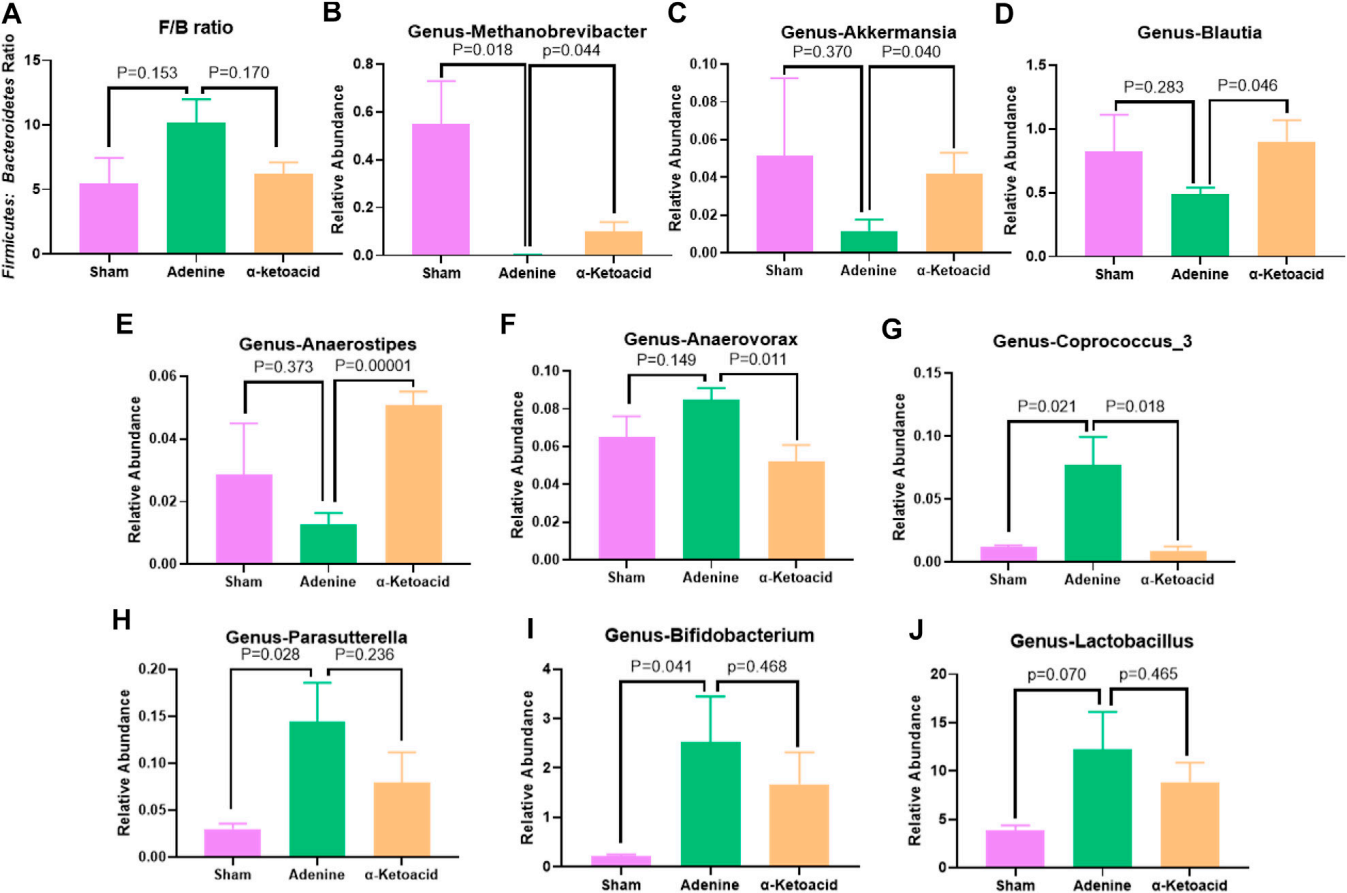
The relative abundances of differential bacteria in the gut microbiota at genus levels. **(A)** *Firmicutes* to *Bacteroidetes* ratio. **(B)** *Methanobrevibacter.* **(C)** *Akkermansia.* **(D)** *Blautia.* **(E)** *Anaerositipes.* **(F)** *Anaerovorax.* **(G)** *Coprococcus_3.* **(H)** *Parasutterella.* **(K)** *Bifidobacterium.* **(I)** *Lactobacillus.* Data are presented as the means ± standard error of the mean. *n* = 8 rats per group. A value of *p* < 0.05 was considered statistically significant by Welch’s *t* test.

We next aimed to estimate the effects of *α*-ketoacid on intestinal microbiota of adenine-induced CKD rats. The Chao and ace index were calculated to examine the *α*-diversity metrics. The differences between Sham Group and Adenine Group were significant in the Chao index (*p* = 0.03, Figure 3A) and ace index (*p* = 0.02, Figure 3B) was significantly, but there was no significant difference in alpha diversity between Adenine Group and *a*-ketoacid Group (*p* = 0.99 for Chao; *p* = 0.91 for ace). However, based on unweighted unifrac distances, the PCoA visually revealed that the microbiota structure of Adenine Group clearly separated from than of Sham group and *α*-ketoacid Group (Figure 3C). Furtherly, the relative abundances of differential bacteria in the gut microbiota at phylum level and genus level was analyzed (Figures 3D,E). At the phylum level, phylum *Firmicutes* and *Bacteroidetes* were most abundantly presented which accounted for the majority of the population in three groups. Compared to Sham group, the *Firmicutes* phylum was obviously decreased (*p* = 0.01) while *Bacteroidetes* increased in Adenine Group (*p* = 0.07), but there was no difference between Adenine Group and *α*-ketoacid Group (*p* > 0.05). After unclassified microbiota were excluded, F/B ratio was calculated as the ratio between the intestinal abundance of *Firmicutes* and that of *Bacteroidetes* at the genus level (Figure 4A). Compared with Sham group, the ratio of *Firmicutes* to *Bacteroidetes* was increased (*p* = 0.153) while this ratio in *a*-ketoacid Group was lower than Adenine Group (*p* = 0.170). *α*-ketoacid significantly increased the abundance of *Methanobrevibacte*r, *Akkermansia*, *Blautia* and *Anaerositipes* (*p* = 0.044 for *Methanobrevibacte*r, *p* = 0.040 for *Akkermansia*, *p* = 0.046 for *Blautia* and *p* = 0.00001 for *Anaerositipes*) (Figures 4B–E). The relative abundance of *Anaerovorax* and *Coprococcus_3* in *a*-ketoacid Group was significantly lower than that in Adenine Group (*p* = 0.011 for *Anaerovorax*, *p* = 0.018 for *Coprococcus_3*) (Figures 4F,G). The relative abundance of *Parasutterella* in Adenine Group was significantly lower than that in Sham group (*p* = 0.028), while *α*-ketoacid intervention tended to increase the abundance of *Parasutterella* (*p* = 0.236) (Figure 4H). *α*-Ketoacid could partly restore the abnormal relative abundances of above bacteria in CKD rats at genus level. It seemed that more *Bifidobacterium* and *Lactobacillus* were colonized in the intestines of adenine-induced CKD rats (*p* = 0.041 for *Bifidobacterium*, *p* = 0.070 for *Lactobacillus*) (Figures 4I,J).

KEGG function analysis with Tax4Fun by Kruskal-Wallis showed that Lysine biosynthesis, Lysine degradation, *Glycine*, serine and threonine metabolism, Histidine metabolism, Tyrosine metabolism, Valine, leucine and isoleucine degradation, Tryptophan metabolism were significantly different among groups (Supplementary Figure S2).

### *α*-Ketoacid Altered Serum Metabolite Profiles on Uremic Toxins and Bile Acids in Chronic Kidney Disease Rats

#### Global Metabolic Profiling of Serum Metabolites in Adenine-Induced Chronic Kidney Disease Rats

**FIGURE 5:**
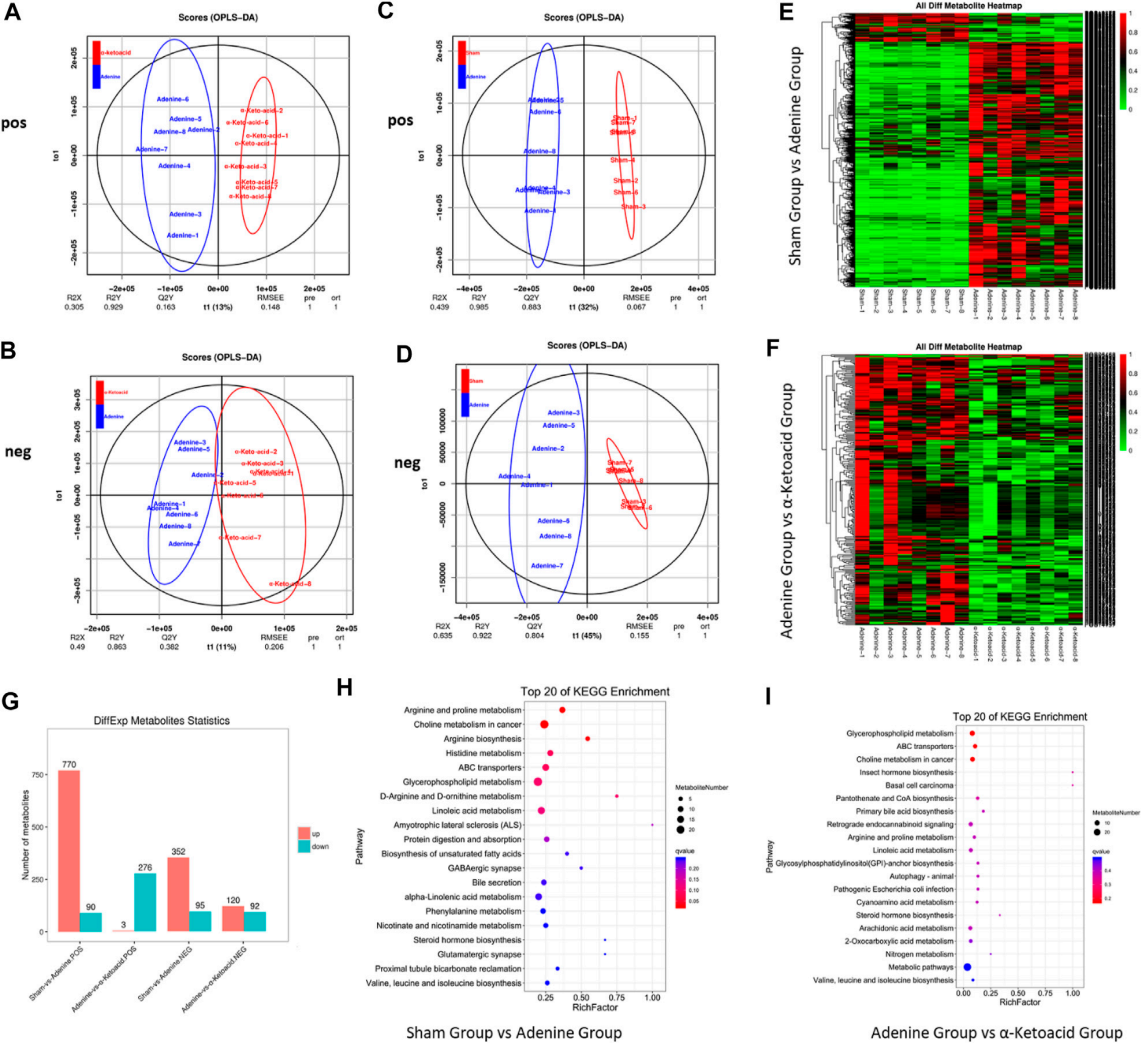
α-ketoacid altered serum metabolite profiles in adenine-induced CKD rats. Principle component analysis score plots of serum metabolites: comparison between Adenine and *α*-ketoacid Group in positive ion **(A)** and negative ion **(B)** modes. Principle component analysis score plots of serum metabolites: comparison between Sham and Adenine Group in positive ion **(C)** and negative ion **(D)**. **(E)** Heatmap of differential metabolites between Sham and Adenine Group. **(F)** Heatmap of differential metabolites between Sham and Adenine Group. **(G)** Number of differential metabolites Adenine and *α*-ketoacid Group. **(E)** Bubble plots of KEGG pathway enrichment analysis between Sham and Adenine Group and between Adenine and *α*-ketoacid Group. Welch’s *t* test.

A total of 1921 peaks in the negative mode and 3,471 peaks in the positive mode were identified for further analysis. In OPLS-DA analysis, the score plots exhibited distinct populations between Sham and Adenine Group (Figures 5A,B). as well as between Adenine Group and *α*-ketoacid Group (Figures 5C,D). Heatmaps suggested that *α*-ketoacid significantly reshaped the metabolic patterns of serum metabolites in adenine-induced CKD rats in both positive and negative modes (Figures 5E,F). Based on the OPLS-DA models, metabolites with a variable importance value >1, fold-change ≥ 1.2 or ≤0.83, and q-value < 0.05 were defined as differential metabolites among the groups. Between Sham Group and Adenine Group, 770 upregulated and 90 downregulated metabolites in positive ion mode and 352 upregulated and 95 downregulated metabolites in negative ion mode with significant changes in peak intensity were detected. On the other hand, three upregulated and 276 downregulated metabolites in positive ion mode and 120 upregulated and 92 downregulated metabolites in negative ion mode were detected between Adenine Group and *α*-Ketoacid Group (Figure 5G).

To explore the functional significance of the serum metabolic changes in *α*-Ketoacid Group, metabolic pathway enrichment analysis of differential metabolites was carried out using the KEGG database. Metabolic pathways closely related to *α*-ketoacid were figured out, including glycerophospholipid metabolism, ABC transporters, choline metabolism in cancer, insect hormone biosynthesis, basal cell carcinoma, pantothenate and CoA biosynthesis, primary bile acid biosynthesis (Figures 5H,I).

#### *α*-Ketoacid Altered Serum Metabolite Profiles on Uremic Toxins in Adenine-Induced Chronic Kidney Disease Rats

**FIGURE 6:**
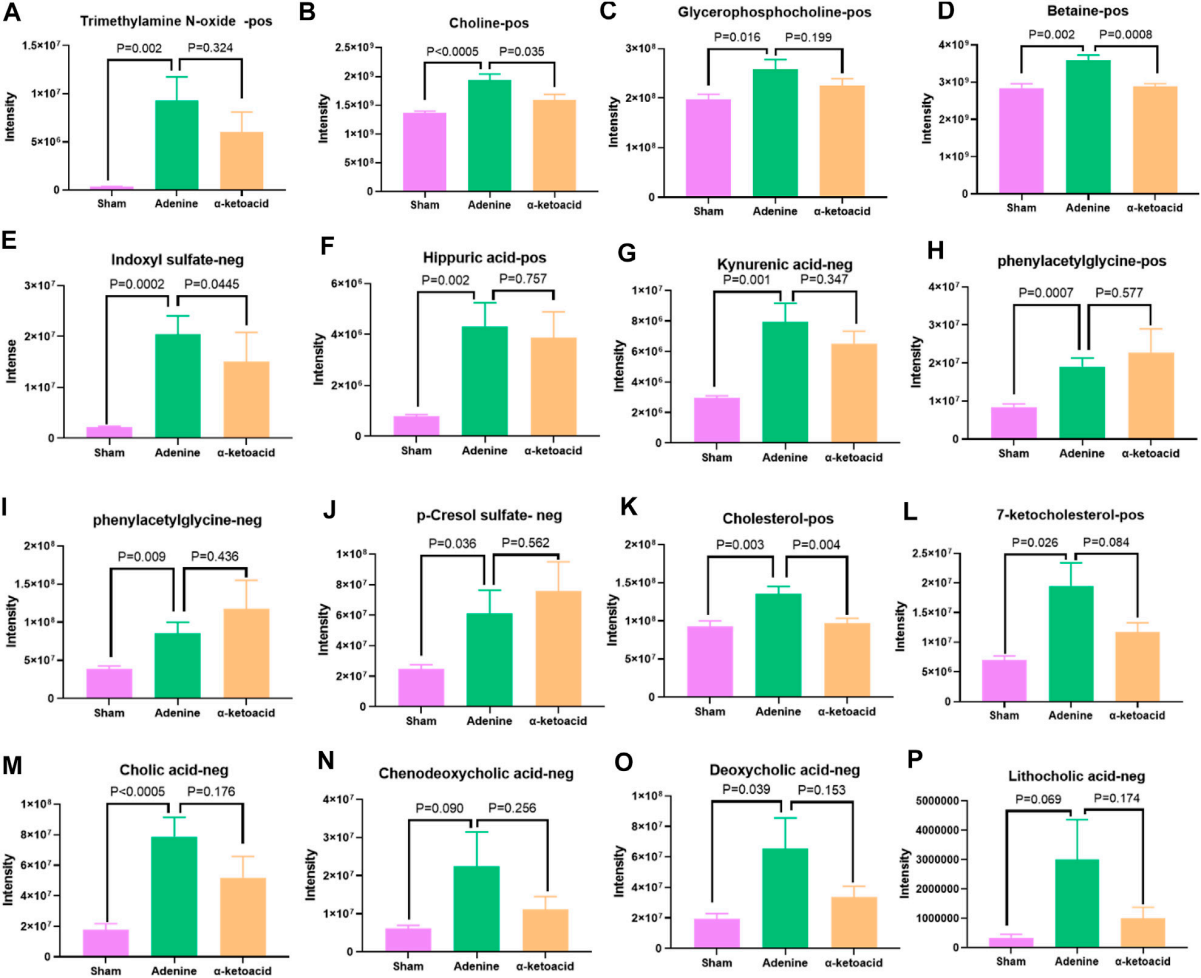
α-ketoacid altered serum metabolites of uremic toxins and bile acids in adenine-induced CKD rats. **(A)**trimethylamine N-oxide. **(B)** choline. **(C)** phosphatidylcholine. **(D)** betaine. **(E)** indoxyl sulfate. **(F)** hippuric acid. **(G)** kynurenic acid. **(H)** phenylacetylglycine. **(I)** phenylacetylglutamine. **(J)** p-cresol sulfate. **(K)** cholesterol**. (L)** 7-ketocholesterol. **(M)** cholic acid. **(N)** chenodeoxycholic acid. **(O)** deoxycholic acid. **(** ***P*** **)** lithocholic acid. Data are presented as the means ± standard error of the mean. *n* = 8 rats per group. A value of *p* < 0.05 was considered statistically significant by Welch’s *t* test.

Metabolic pathway enrichment analysis of differential metabolites based on the KEGG database identified glycerophospholipid metabolism and choline metabolism, which are both related to the production of trimethylamine (TMA)/trimethylamine N-oxide (TMAO). Compared with Sham Group, CKD rats in Adenine Group had significantly increased level of TMAO and its precursors including choline, phosphatidylcholine and betaine (*p* = 0.002 for TMAO, *p* < 0.0005 for choline, *p* = 0.016 for phosphatidylcholine and *p* = 0.002 for betaine), which were decreased after *α*-ketoacid treatment (*p* = 0.324 for TMAO, *p* = 0.035 for choline, *p* = 0.199 for phosphatidylcholine and *p* = 0.0008 for betaine) (Figures 6A–D).

Besides TMAO, we also focused on other uremic toxins. Compared with Sham Group, indoxyl sulfate (IS), hippuric acid and kynurenic acid were elevated in Adenine Group (*p* = 0.0002 for IS, *p* < 0.0005 for hippuric acid and *p* = 0.001 for kynurenic acid). *α*-ketoacid could have the trend to reduce the level of above uremic toxins (*p* = 0.0445 for IS, *p* = 0.757 for hippuric acid and *p* = 0.347 for kynurenic acid) (Figures 6E–G). Adenine Group had significantly higher level of phenylacetylglycine (PAGly), phenylacetylglutamine (PAGln) and p-cresol sulfate (PCS) than Sham Group (*p* = 0.0007 for PAGly, *p* = 0.009 for PAGIn, *p* = 0.036 for PCS). However, CKD rats tend to produce more PAGly, PAGln, and PCS after *α*-ketoacid treatment (Figures 6H–J).

#### *α*-Ketoacid Altered Serum Metabolite Profiles on Bile Acids in Adenine-Induced Chronic Kidney Disease Rats

It was reported that TMAO could reduce reverse cholesterol transport in the bile acid synthetic pathway and inhibit cholesterol elimination from the body (Koeth et al., 2013). In our study, cholesterol and 7-ketocholesterol increased significantly in Sham rats (*p* = 0.003 for cholesterol, *p* = 0.026 for7-ketocholesterol) and reduced after *α*-ketoacid administration (*p* = 0.004 for cholesterol, *p* = 0.084 for 7-ketocholesterol) (Figures 6K–L).

As for bile acid profiles, the oxidation of cholesterol in the liver synthesizes primary bile acids, including cholic acid (CA) and chenodeoxycholic acid (CDCA) (Lefebvre et al., 2009). Colon microbiota can further metabolize primary bile acids to produce secondary bile acids like deoxycholic acid (DCA) and lithocholic acid (LCA) (Devlin and Fischbach, 2015). In our study, CA, CDCA, DCA and LCA were increased in Adenine-induced CKD rats (*p* < 0.0005 for CA, *p* = 0.090 for CDCA, *p* = 0.039 for DCA, *p* = 0.069 for LCA) and α-ketoacid had the trend to improve the bile acid profiles (*p* = 0.176 for CA, *p* = 0.256 for CDCA, *p* = 0.153 for DCA, *p* = 0.174 for LCA) (Figures 6M–P).

### Correlations Between Microbial Abundances and Metabolic Profiling

**FIGURE 7:**
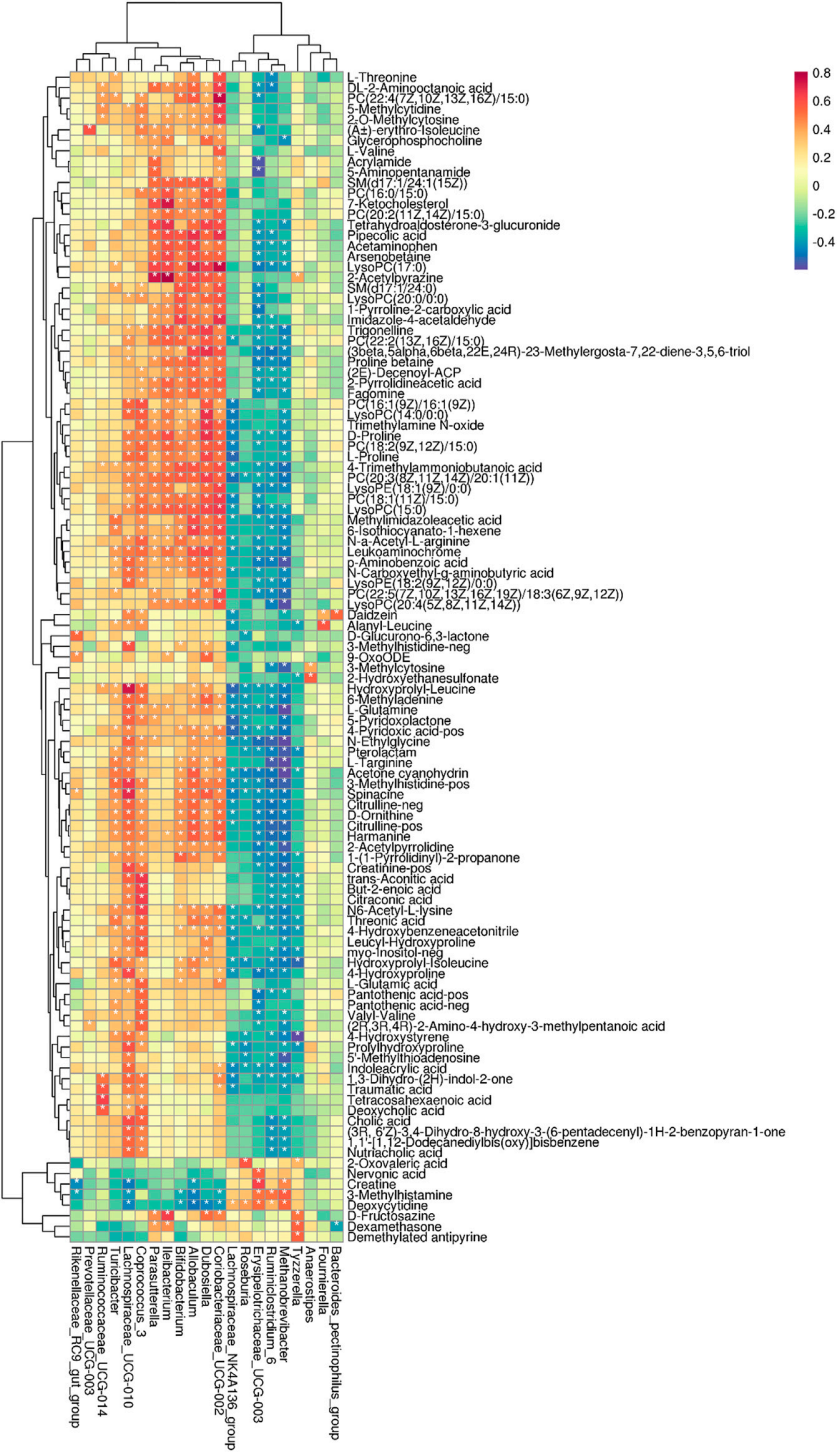
Correlations between microbial abundances in the genus level and metabolic profiling. *p < 0.05.

Finally, to further dig out the correlations between serum metabolites (VIP≥1 and *t*-test *p* < 0.05) and intestinal microbiome (Welch’s T text *p* < 0.05), Spearman’s correlation analysis was conducted to reveal the potential relationships between serum metabolites and microbial abundances at the genus level. The coefficients of correlation between potential biomarkers highly associated with *α*-ketoacid treatment and relative abundances of gut microbiota at the genus level are shown in a heatmap (Figure 7).

The correlation analysis revealed that Ruminococcaceae_UCG-014 was positively correlated with betaine and DCA (*p* < 0.05). Dubosiella was positively correlated with TMAO, Glycerophosphocholine, and 7-Ketocholesterol. Lachnospiraceae_UCG-010 was positively correlated with creatinine, hippuric acid and CA. Methanobrevibacter was negatively correlated with creatinine. Parasutterella was positively correlated with 7-Ketocholesterol. Coriobacteriaceae_UCG-002 was positively correlated with TMAO. Coprococcus_3 was positively correlated with betaine, TMAO, IS, CA, and DCA (Figure 7). Collectively, these results indicated that the changes in the colonic microbiota were correlated with alterations of metabolites in adenine-induced CKD rats after *α*-ketoacid treatment.

## Discussion

α-Ketoacid is a commonly used prescription medicine for ESKD patients to supplement with a LPD (Mitch, 2000; Bernhard et al., 2001). In the present study, we investigated the response of the colonic microbes and serum metabolites of adenine-induced CKD rats fed *α*-ketoacid using biochemical analyses, 16S rRNA sequencing, and UPLC-MS/MS. Our results showed that *α*-ketoacid treatment markedly reduced serum BUN, SCR level and 24 h urine protein, and alleviated tubular atrophy, glomerulosclerosis and interstitial fibrosis in adenine-induced CKD rats. *α*-Ketoacid treatment also had a tendency to alleviate abnormal weight loss. Moreover, *α*-ketoacid significantly increased the abundance of *Methanobrevibacter, Akkermansia*, *Blautia* and *Anaerositipes* while reduced the abundance of *Anaerovorax* and *Coprococcus_3* at the genus level. In addition, our results also demonstrated that *α*-ketoacid significantly reduced the concentrations of indoxyl sulfate, betaine, choline and cholesterol. These findings indicated *α*-ketoacid had a marked influence on the intestinal microbiota and serum metabolic profiles in CKD rats.

Our result of *α*-ketoacid in protecting against CKD are in line with some animal and human random controlled trials, which have showed the role of *α*-ketoacid supplemented with protein restriction in improving kidney function, reducing proteinuria, and alleviating histological damage (Koppe et al., 2019). Such reno-protective effect may be explained by the reduction of protein intake. Brenner et al. have shown that high protein intake causes obvious renal hypertrophy, inducing high glomerular pressure and ultrafiltration, which poses negative effects on kidney function (Brenner et al., 1982). Marzocco et al. also reported that IS level was determined majorly by higher protein intake (Marzocco et al., 2013). On the other hand, studies suggest that restricted LPD combined with *α*-ketoacid may improve renal function and nutritional status, while alleviate hyperparathyroidism, insulin resistance, and reduce the accumulation of uremic retention solutes (Koppe et al., 2019). The main concern in the studies mentioned is the fact that *α*-ketoacids are not given solely but combined with low protein intake restriction. Our study is to discover a supplementation of *α*-ketoacid alone in normal diet condition without low protein intake restriction could delay the progression of CKD. Our study also showed that *α*-ketoacid could improve the intestinal barrier. The intestinal physical barrier function of the intestine depends on the mucosal integrity maintained by the cellular tight junctions among intestinal epithelial cells (Kurashima and Kiyono, 2017). The disturbance of the expression of the tight junction protein including ZO-1, Occludin and Claudin-1 means damage of intestinal of intestinal mucosal structure, which may increase the permeability of intestinal epithelium and lead to bacterial translocation that was accompanied by increasing level of DAO in serum (Li et al., 2017). The enzyme DAO is mainly expressed in intestinal mucosa and usually has a low presence in blood. The amount of DAO is positively associated with the permeability of the intestinal barrier (Wolvekamp and de Bruin, 1994). Our present study exhibited orally administrated *α*-ketoacid could improve the intestinal physical barrier function by modulating mucosal structures and up-regulating the expression of tight junction proteins, thereby reducing the level of DAO translated from colon to serum. *α*-Ketoacid also could regulate the intestinal immunological barrier function by stimulating the production of IL-22, whose key role in mucosal defense is known to promote epithelial cell regeneration and antimicrobial peptide production (Yang et al., 2020). Simultaneously, *α*-ketoacid could differentially impact the composition and metabolism of microbiota that we discussed in detail below.16s rRNA gene sequencing was carried out to investigate the impact of *α*-ketoacid treatment on gut microbiota in adenine-induced CKD rats. We found that the *α*-ketoacid reduced the bacterial richness and diversity, as indicated by the Chao values and ace index. The diversity of Adenine Group was higher than that of Sham Group, probably due to the overgrowth of pathogenic bacteria (Ji et al., 2020) and *α*-ketoacid Group tended to correct the abnormity. As for *β*-diversity metrics, the results of PCoA analyses further revealed difference in the colonic bacterial communities among the three groups. In the phylum level analysis, the Bacteroidete and Firmicutes were the two major microbiome members, which play crucial roles in modulating host inflammation and immune balance (Chang et al., 2015). Moreover, the elevation of the F/B ratio is positively related with gut permeability (Robertson et al., 2017). In our study, we could observe F/B ratio was higher in Adenine Group than that in Sham group and decreased in *α*-ketoacid Group. The difference of F/B ration among groups were not significant, but the trend was similar with the change of intestinal barrier, which were also in agreement with the findings that demonstrated higher *Firmicutes* to *Bacteroidetes* ratio in patients and animals with obesity (Wang et al., 2018) and hypertension (Mushtaq et al., 2019).

At the genus level, we found the enrichment of *Methanobrevibacter, Akkermansia*, *Blautia* and *Anaerositipes*, and the depletion of *Anaerovorax* and *Coprococcus_3* in our CKD rats after *α*-ketoacid treatment. *Methanobrevibacte*r acts as an “power broker” in the distal gut flora, regulating the specificity of polysaccharide fermentation and affecting the calorie deposition in fat storage (Samuel and Gordon, 2006), which perhaps is the reason that the level of cholesterol was significantly reduced after *α*-ketoacid treatment. It has been reported that the proportions of the genus Akkermansia levels was significantly higher in the athletes and low BMI individuals than in the high BMI group (Clarke et al., 2014). Oral administration of *Akkermansia* can increase the number of goblet cells, restore the mucus thickness of the inner layer, and up-regulated the expression of tight-junction proteins including occludin, claudin, and ZO-1, in the gut of mice with metabolic syndrome (Everard et al., 2013; Grander et al., 2018). Intestinal barrier is often impaired in patients with ESKD (Vaziri et al., 2016). Improved gut barrier function could reduce leakage of bacterial wall products, bacterial metabolites, and live bacteria into the circulation, thereby inhibiting local and systematic inflammatory response (Meijers et al., 2019). The increasing abundance of *Blautia* in our animal study was similar to the alterations of the human gut microbiome in CKD (Ren et al., 2020). In previous studies, genus *Blautia* is identified to produce SCFAs and positively correlated with metabolic homeostasis improvement (Bai et al., 2018; Tong et al., 2018). *Anaerostipes* is a kind of butyrate-producing bacteria. As one of the most important metabolites of the gut microbiota for host health, butyrate provides the preferential energy source of intestinal epithelium, stimulates the production of regulatory T cells and inhibits inflammation (Zhang et al., 2016). We also found that the relative abundance of *Parasutterella* in Adenine Group was significantly lower than that in Sham group, while *α*-ketoacid intervention tended to increase the abundance of *Parasutterella*. In a variety of animal models and human studies, a marked decrease of *Parasutterella* has been observed after high-fat diet (HFD) treatment, indicating a negative correlation was existed between the abundance of *Parasutterella* and HFD-induced metabolic abnormality (Zhang et al., 2012; Danneskiold-Samsoe et al., 2017). As for *Anaerovorax*, it has been approved as butyrate producers (Liu et al., 2019). However, the effect of decreasing *Anaerovorax* on SCFA after *α*-ketoacid intervention presumably be weaker than that of other SCFA-producing microbiota. Therefore, the total effect of *α*-ketoacid is repairing intestinal barrier in Adenine-induced CKD rats. Coprococcus_3 is one of the main genera that differed between the patients with idiopathic membranous nephropathy and healthy groups (Lang et al., 2020). It is also reported that the genera of Coprococcus three in the ileum were correlated positively with lipid metabolic dysfunction and pro-inflammatory response in low-birth-weight pigs (Huang et al., 2020), which is in accord with our finding that abundance of Coprococcus_3 was positively correlated with serum level of TMAO and IS. Therefore, these findings indicated that *a*-ketoacids can promote the abundance of a variety of probiotic such as SCFA-producing bacteria while inhibit the abundance of potential pathogens, which may also pose beneficial effects on CKD rats. It is noted that more *Bifidobacterium* and *Lactobacillus* were colonized in the intestines of adenine-induced CKD rats, which is similar to the study by Liu et al. (Liu et al., 2021). *Bifidobacterium* and *Lactobacillus* are the most common probiotics in the intestinal tract, which have important health-promoting functions. We believe that, in response to the effect of CKD on intestines, the body multiplies many probiotics, which stimulates the growth of other microorganisms while effectively regulating mucosal and immune responses. Another reason may be that the interaction between the symbiotic bacteria and the host is quite complex and may show beneficial or harmful properties under certain circumstances. For example, *lactobacillus reuteri* colonizes lupus-prone hosts and translocates to mesenteric lymph nodes, liver, and spleen, and exacerbates TLR7-dependent lupus in conventional and germ-free mice.

In the gut community, differences in microbiota population also can bring about different microbial metabolic process and metabolite profiles. In our study, OPLS-DA analyses showed a clear separation of serum metabolites among groups, indicating significant differences in the metabolic profiles. To investigate the potential mechanisms of *α*-ketoacid, metabolic pathway enrichment analysis of differential metabolites based on the KEGG database identified seven metabolic pathways, including glycerophospholipid metabolism and choline metabolism, both of which are related to the production of TMA/TMAO. TMAO is a kind of uremic toxin that formed in the liver from TMA, which is produced by the action of gut microbiota using dietary precursors, including phosphatidylcholine, choline, and betaine (Wang et al., 2011; Tang et al., 2013). Compared with Sham Group, TMAO and its precursors were increased significantly in Adenine Group, while *α*-ketoacid treatment could decrease the serum level of TMAO, phosphatidylcholine, choline and betaine to some different contents. Elevated plasma levels of TMAO and its precursors are each associated with poor prognosis of CKD Gupta et al. (2020) and cardiovascular disease (Wang et al., 2011; Wang et al., 2014). It has been known that TMAO alters the enterohepatic cholesterol metabolism, reducing cholesterol eliminating from the body by inhibiting cholesterol reverse cholesterol transport (Koeth et al., 2013; Warrier et al., 2015). In our study, the level of cholesterol and 7-keto-cholesterol was reduced after *α*-ketoacid treatment. Formed by oxygen radical attack on cholesterol, 7-keto-cholesterol is the most abundant oxysterol found in the blood and arterial plaques of patients with coronary artery disease (Anderson et al., 2020).

Primary bile acid biosynthesis is another differential metabolite enrichment pathway. Bile acids are synthesized from cholesterol in the liver, stored in the gallbladder, and delivered into the intestine after meals (Lefebvre et al., 2009). In recent years, bile acids have been considered to play a role like hormones to regulate a variety of metabolic processes. Importantly, secondary bile acids are synthesized by gut microorganisms from primary bile acids. Intestinal flora can regulate bile acid pools and bile acid receptors in intestinal tract, thereby triggering various metabolic pathways and affecting host metabolism. Bile acids, in turn, alter the composition of gut flora (Fiorucci and Distrutti, 2015). Our results indicated that *α*-ketoacid could affected cholesterol metabolism and the levels of serum primary bile acids in adenine-induced CKD rats. In addition, the level of secondary bile acids was abnormal in CKD rats and reversed by *α*-ketoacid treatment, which might be related with the change of intestinal flora. Here, the level of bile acid in Adenine Group was higher than that in Sham Group, which has been identified as a metabolic indicator of abnormal lipid metabolism in diabetes rats (Yan et al., 2020) and a mediator of intestinal microbiome to aggravate ESKD (Wang et al., 2020).

We also focused on protein-bound uremic toxin. In our study, *α*-ketoacid reduced the level of IS significantly and had the trend to reduce the level of hippuric acid and kynurenic acid, concurred with the results of two prospective, randomized, crossover-controlled trials, which reported that VLPD supplemented with ketoanalogues reduced IS and PCS serum levels in CKD patients compared with LPD (Di Iorio et al., 2012) and free diet (Rocchetti et al., 2021). However, CKD rats tend to produce more PAGly, PAGln and PCS after *α*-ketoacid treatment. After ingestion of phenylalanine, most essential amino acids are absorbed in the small intestines, but the unabsorbed phenylalanine that reaches the large intestine can be metabolized by intestinal flora to form phenylpyruvate (the deammoniation product produced by the initial microbiota) and subsequently phenylacetic acid. After absorption into the portal system, phenylacetic acid is readily metabolized in the liver to produce PAGLn (the main product in humans) and PAGly (the main product in mice). Another uremic toxin, PCS, which is also highly conjugated with proteins, is a product of the intestinal bacterium metabolism of tyrosine and phenylalanine. PAGly and PAGln were shown to enhance platelet activation-related phenotypes and thrombotic potential in animal models with arterial injury (Nemet et al., 2020). PCS have been experimentally proved to cause renal tubular damage and induce renal fibrosis in CKD rats (Vanholder et al., 2014). We should notice that *α*-ketoacid has the potential to increase the uremic toxins produced by the metabolism of phenylalanine and tyrosine, which both are compositions of *α*-ketoacid. Furthermore, an observational study found that a lower dose of *α*-ketoacid was more beneficial in slowing the deterioration of renal function in patients with ESKD (Chen et al., 2012). Another study suggested that amino acid supplements like lysine may pose protective effect against vascular calcification (Shimomura et al., 2014). In our study, as precursors of uremic toxins, phenylalanine and tyrosine seems to be deleterious after *α*-ketoacid treatment. Therefore, a question about that what are the optimal dose and composition of *α*-ketoacid that are needed to put forward and further study.

Our microbiome-to-metabolome association study identified that the abundance of Coprococcus_3 was positively correlated with serum level of betaine, TMAO, IS, DCA, and CA, which might suggest that microbes involved in uremic toxin and bile acid production might be used as therapeutic target for ESKD. However, the result attained by Spearman’s correlation analysis only demonstrated the correlational relationship Coprococcus_3 and serum level of betaine, TMAO, IS, DCA and CA but not causal relationship between. Further studies like fecal microbiota transplantation are needed to discover the potential mechanism how Coprococcus_3 affecting the metabolism of TMAO, IS, DCA, and CA.

## Conclusion

By integrating sequencing of gut microbiota and serum untargeted metabolomics, *α*-ketoacid treatment was observed to improve kidney function, reduce proteinuria, and alleviate histological damage of kidney in adenine-induced CKD rats. In addition, *α*-ketoacid played an important role in modifying gut microbiome, improving the intestinal barrier, altering serum metabolite profiles on uremic toxins and bile acids. These findings may help us to have a comprehensive of understand on the effects of *α*-ketoacid on CKD, suggesting that *α*-ketoacid treatment may be included as an essential part of the clinical recommendations not only for both the nutritional prevention, but also for metabolic management of CKD.

## Data Availability

The datasets presented in this study can be found in online repositories. The names of the repository/repositories and accession number(s) can be found below: https://www.ncbi.nlm.nih.gov/, PRJNA688355.

## Abbreviations

BUN, blood urea nitrogen; CA, cholic acid; CDCA, chenodeoxycholic acid; CKD, chronic kidney disease; DCA, deoxycholic acid; ESKD, end-stage kidney disease; HE, hematoxylin and eosin; HFD, high-fat diet; IS, indoxyl sulfate; KEGG, Kyoto Encyclopedia of Genes and Genomes; LCA, lithocholic acid; LC-MS/MS, liquid chromatography-tandem mass spectrometry; LPD, low protein diet; OPLS-DA, orthogonal partial least squares discrimination analysis; OTUs, operational taxonomic units; PAGly, phenylacetylglycine; PAGln, phenylacetylglutamine; PCS, p-cresol sulfate; PCoA, principal coordinate analysis; SCR, serum creatinine; TMA, trimethylamine; TMAO, trimethylamine N-oxide; UHPLC, Ultra-high performance liquid chromatography; VLPD, very low protein diet.
