# Supplementary material for: Microbiome-Metabolomics Analysis Reveals the Protection Mechanism of α-Ketoacid on Adenine-Induced Chronic Kidney Disease in Rats
Source: Front Pharmacol. 2021 May 11;12:657827. doi: 10.3389/fphar.2021.657827 (PMC8144710; doi:10.3389/fphar.2021.657827)
Supplement: Supplementary file 1 [file DataSheet1.docx]

Supplementary Material

**Supplementary Item 1. instruments and regents, metabolite extraction, data preprocessing and annotation, statistical analysis of UPLC–MS analysis for metabolomics.**

1. **Regents**

| Regents | Source | CAS |
| --- | --- | --- |
| Methanol | CNW Technologies | 67-56-1 |
| Ammonium acetate | CNW Technologies | 631-61-8 |
| Formic acid | CNW Technologies | 64-18-6 |
| Acetonitrile | CNW Technologies | 75-05-8 |
| Ammonium hydroxide | CNW Technologies | 1336-21-6 |
| Internal standard:  2-Chloro-L-henylalanine | Shanghai Hengbai Biotech C., Ltd. | 103616-89-3 |

1. **Instruments**

| Instrument | Model | Source |
| --- | --- | --- |
| UHPLC | 1290 UHPLC | Agilent |
| Mass Spectrometer | Q Exactive Orbitrap | Thermo Fisher Scientific |
| Centrifuge | Heraeus Fresco17 | Thermo Fisher Scientific |
| Scales | BSA124S-CW | Sartorius |
| Water Purification | Ming che D24 UV | Merck Millipore |
| Column | ACQUITY UPLC HSS T3 (2.1 × 100 mm, 1.7 μm) | Waters |

1. **Metabolites extraction**
2. Methanol (300 µL) and internal standard (20uL) was added to serum (100 µL) and vortex-mixed vigorously for 3min. The mixture was settled at room temperature for 10min, followed by incubation at -20℃for 1 h and centrifugation at 12000 rpm and 4℃ for 15 min. The resulting supernatants were transferred to LC-MS vials and stored at -80℃ until the LC-MS/MS analysis. The quality control (QC) sample was prepared by mixing an equal aliquot of the supernatants from all of the samples.**LC-MS/MS analysis**

LC-MS/MS analyses were performed using an UHPLC system (1290, Agilent Technologies) with a UPLC HSS T3 column (2.1 mm × 100 mm, 1.7 μm) coupled to Q Exactive (Orbitrap MS, Thermo). The mobile phase A was 0.1% formic acid in water for positive, and 5 mmol/L ammonium acetate in water for negative, and the mobile phase B was acetonitrile. The elution gradient was set as follows: 0 min, 1% B; 1 min, 1% B; 8 min, 99% B; 10 min, 99% B; 10.1 min, 1% B; 12 min, 1% B. The flow rate was 0.5 mL/min. The injection volume was 2 μL. The QE mass spectrometer was used for its ability to acquire MS/MS spectra on an information-dependent basis (IDA) during an LC/MS experiment. In this mode, the acquisition software (Xcalibur 4.0.27, Thermo) continuously evaluates the full scan survey MS data as it collects and triggers the acquisition of MS/MS spectra depending on preselected criteria. ESI source conditions were set as following: Sheath gas flow rate as 45 Arb, Aux gas flow rate as 15Arb, Capillary temperature 320℃, Full ms resolution as 70000, MS/MS resolution as 17500, Collision energy as 20/40/60 eV in NCE model, Spray Voltage as 3.8 kV (positive) or -3.1 kV (negative), respectively.

1. **Data preprocessing and annotation**

MS raw data(.raw) files were converted to the mzML format using ProteoWizard, and processed by R package XCMS (version 3.2), including retention time alignment, peak detection and peak matching. Then the data were filtered by the followed criterion: sample numbers contains a metabolite was less than 50% all sample numbers in a group (QC were also taken as a group). Normalization to an internal standard for each sample was done subsequently. Next, missing values were replaced by the half of the minimum value found in the dataset by default. The preprocessing results generated a data matrix that consisted of the retention time (RT), mass-to-charge ratio (m/z) values, and peak intensity.

OSI-SMMS (version 1.0, Dalian Chem Data Solution Information Technology Co. Ltd.) was used for peak annotation after data processing with in-house MS/MS database.

1. **Multivariate statistical analysis.**

**OPLS-DA:** Orthogonal projection to latent structures-discriminant analysis (OPLS-DA). OPLS-DA is an extension of PLS-DA which incorporates an Orthogonal Signal Correction (OSC) filter into a PLS model. The basic concept in OPLS is to separate the systematic variation in X into two parts, one that is correlated to Y and one that is not correlated (orthogonal) with Y. Only the Y‐predictive variation is used to model the data. OPLS-DA was applied in comparison groups using R package models (http://www.r-project.org/). The OPLS-DA model was further validated by cross-validation and 200 permutation tests. For cross-validation, the data was partitioned into seven subsets, where each of the subsets was then used as a validation set. R2 indicated the total variation in the data matrix that was explained by the model. Predictive ability (Q2) values represented the most recognized diagnostic statistical parameter to validate the OPLS-DA model in metabolomics. Acceptable predictive model is considered for Q2 value greater than 0.4. Good predictive model is considered for Q2 value greater than 0.9. Permutation test randomly permutes class labels for 200 times and then produces a distribution of R2’ values and Q2’ values. In essence, a reliable model should yield significantly larger R2 and Q2 value compared to R2’ and Q2’ values generated from random models using the same data set.

Differential metabolites analysis: A variable importance in projection (VIP) score of (O)PLS model was applied to rank the metabolites that best distinguished between two groups. The threshold of VIP was set to 1. In addition, T-test was also used as a univariate analysis for screening differential metabolites. Those with a P value of T test＜0.05 and VIP≥1 were considered differential metabolites between two groups.

KEGG pathway analysis: KEGG is the major public pathway-related database that includes not only genes but metabolites. Metabolites were mapped to KEGG metabolic pathways for pathway analysis and enrichment analysis. Pathway enrichment analysis identified significantly enriched metabolic pathways or signal transduction pathways in differential metabolites comparing with the whole background. The calculating formula is as follows:


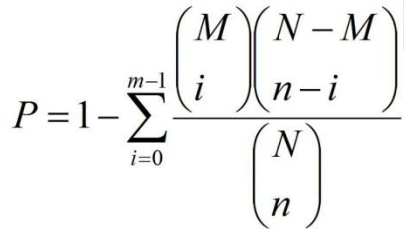


Here N is the number of all metabolites that with KEGG annotation, n is the number of differential metabolites in N, M is the number of all metabolites annotated to specific pathways, and m is number of differential metabolites in M. The calculated p-value was gone through FDR Correction, taking FDR≤0.05 as a threshold. Pathways meeting this condition were defined as significantly enriched pathways in differential metabolites.

Supplementary Fig.1 Effect of α-ketoacid on serum albumin and blood pressure in adenine-induced CKD rats. **(A)** Serum albumin. **(B)** systolic blood pressure. **(C)** diastolic blood pressure.


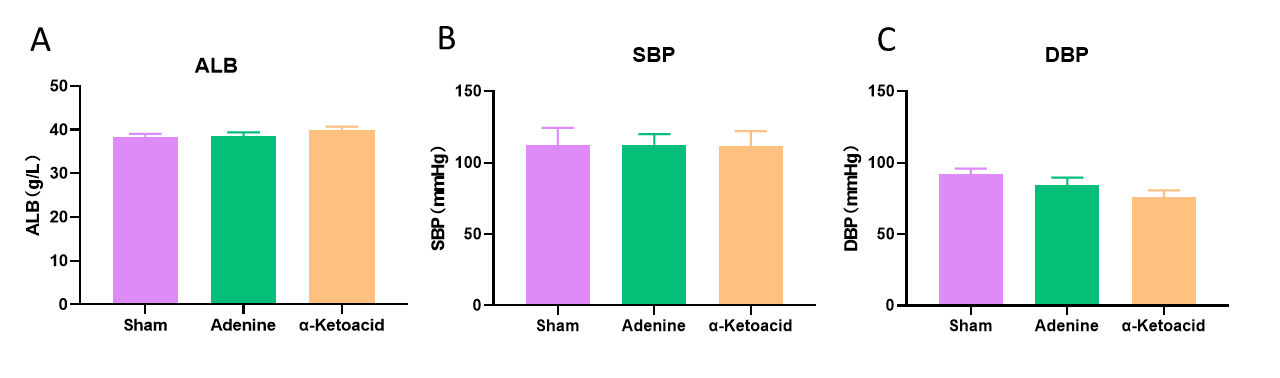


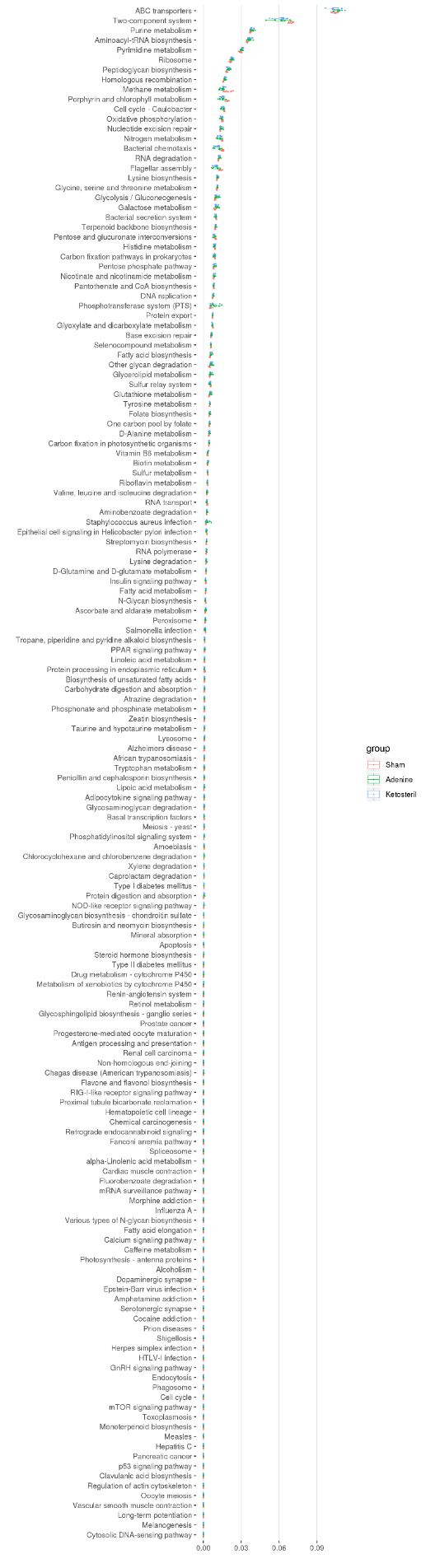
**Supplementary Figure2. KEGG function analysis with Tax4Fun by Kruskal-Wallis.**
